# Wide-Gap Brazing of K417G Alloy Assisted by In Situ Precipitation of M_3_B_2_ Boride Particles

**DOI:** 10.3390/ma13143140

**Published:** 2020-07-14

**Authors:** Zhun Cheng, Xiaoqiang Li, Minai Zhang, Shengguan Qu, Huiyun Li

**Affiliations:** 1 National Engineering Research Center of Near-net-shape Forming Technology for Metallic Materials, South China University of Technology, Guangzhou 510640, China; cheng.zhun@mail.scut.edu.cn (Z.C.); zhang.ma@hotmail.com (M.Z.); qusg@scut.edu.cn (S.Q.); 2 Dongguan Hyperpowder Co. Ltd., Dongguan 523808, China; liyh@hyperpowder.com

**Keywords:** K417G alloy, wide-gap brazed region, M_3_B_2_ borides, tensile strength

## Abstract

In this study, K417G Ni-based superalloy with a 20-mm gap was successfully bonded at 1200 °C using powder metallurgy with a powder mixture. The results indicated that the microstructure and mechanical properties of the as-bonded alloy were highly dependent on the brazing time (15–45 min), mainly due to the precipitation and distribution characteristics of M_3_B_2_ boride particles. Specifically, alloy brazed for 30 min exhibited desirable mechanical properties, such as a high tensile ultimate strength of 971 MPa and an elongation at fracture of 6.5% at room temperature, exceeding the balance value (935 MPa) of the base metal. The excellent strength and plasticity were mainly due to coherent strengthening and dispersion strengthening of the in situ spherical and equiaxed M_3_B_2_ boride particles in the γ + γ′ matrix. In addition, the disappearance of dendrites and the homogenization of the microstructure are other factors that cannot be excluded. This powder metallurgy technique, which can avoid the eutectic transformation of traditional brazing, provides a new effective method for wide-gap repair of alloy materials.

## 1. Introduction

K417G Ni-based superalloy is widely used in the manufacture of aerospace components, such as turbine nozzle guide vanes, integral wheels, and turbine blades, due to its high structural stability and excellent mechanical properties at high temperatures [1,2,3]. However, these aerospace components are often exposed to high-temperature and high-pressure environments, which may cause microcracks and reduce their service life [4]. In recent years, this issue has been addressed using transient liquid phase (TLP) bonding [5], which is an excellent brazing technique with a filler metal based on eutectic transformations. Since this technique is more efficient and cost-effective compared to other repair techniques, it has been used for bonding aerospace components and repairing cracks [6,7,8]. However, due to the complete melting of the filler metal and the formation of a brittle eutectic structure, it is limited to gaps smaller than 1.5 mm [9,10,11].

With the rapid development of current technology, the maintenance of turbine blades is becoming more and more rigorous [12]. As a result, relatively large cracks or worn areas (>1.5 mm) are discovered in aerospace components, where the narrow gap TLP bonding technique would be inadequate [13]. For wide-gap brazing, the selection and design of the filler metal composition is the key factor to improve the brazing performance [14,15,16,17,18,19]. Considerable effort has been devoted to adding a second gap-filler powder, (hereafter termed additive powder) of similar composition to that of the base metal (BM), to the joint gap, for use with braze powder during the high-temperature brazing process [14,20,21]. During the entire wide-gap brazing process, additive powder with a high melting point remains largely unmelted, thereby providing the necessary capillary force to retain the molten braze powder that would otherwise be too fluid to bridge the faying gap surfaces [16,17]. However, the formation of hard and brittle eutectic structures with uneven distribution cannot be avoided due to their sensitivity to the chemical composition of the filler metal, brazing temperature, and brazing time [22,23,24,25].

We previously reported [26] the successful design of a new type of Ni-based mixed filler powder without eutectic transformation, which can be used in the repair of K417G alloy with a wide gap of 20 mm by introducing in situ precipitated borides. In the process, the high-melting-point additive powder with a supporting function can split the large gap into tiny virtual gaps, while the low-melting-point braze powder with a filler function can be fully melted, for wetting and filling the numerous tiny gaps within the large gaps. However, the effect of the brazing time on the properties of the wide-gap brazing joints and the strengthening mechanisms are not clear. In this study, we analyzed the microstructure, and mechanical properties of K417G alloy brazed repairs by vacuum hot-pressing for different brazing time. In particular, the precise regulation of the shape and distribution of the M_3_B_2_ boride phase inside the wide-gap brazed (WGB) region were investigated, ultimately achieving a high-quality brazing repair of the wide gap of K417G alloy.

## 2. Materials and Methods

### 2.1. Materials

The K417G alloy with a size of Φ80 × H20 mm, and chemical composition, as shown in Table 1, was used as the cast base metal (BM). A wide gap with dimensions of L20 mm × W20 mm × H20 mm was machined into the core of the K417G alloy. In order to perform bonding by wide-gap brazing, the high-melting-point additive powder similar in composition to the BM and low-melting-point braze powder were mixed at the weight ratio of 95:5, to obtain the mixed filler powder. The chemical compositions of the base metal, additive powder, braze powder, and mixed filler powder, as shown in Table 1.

**Figure 1:**
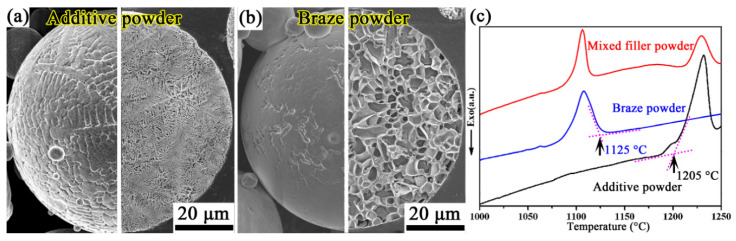
Micrographs of surface morphology and corresponding cross-section of the additive powder (**a**) and braze powder (**b**), and DSC curves of the additive powder, braze powder and mixed filler powder (**c**) (note, the cross-section of the additive powder and braze powder was etched by the prepared corrosion solution (20 g anhydrous CuSO_4_ + 100 mL HCl + 5 mL H_2_SO_4_ + 10 mL H_2_O) for 40 s).

Micrographs of surface morphology and its corresponding cross-section of the additive powder and braze powder are displayed in Figure 1a,b. The spherical additive powder containing dendrites has a melting peak in the high-temperature region, while the spherical braze powder containing cellular crystals has a melting peak in the low-temperature region. As was expected, the mixed filler powder achieved melting peaks both in the high-temperature and low-temperature regions (Figure 1c). Taken together with previous research results [26], the suitable brazing temperature was determined to be 1200 °C, at which the braze powder was completely melted during the brazing process, while the additive powder does not undergo significant melting and continues its supporting role.

### 2.2. Brazing Method

**Figure 2:**
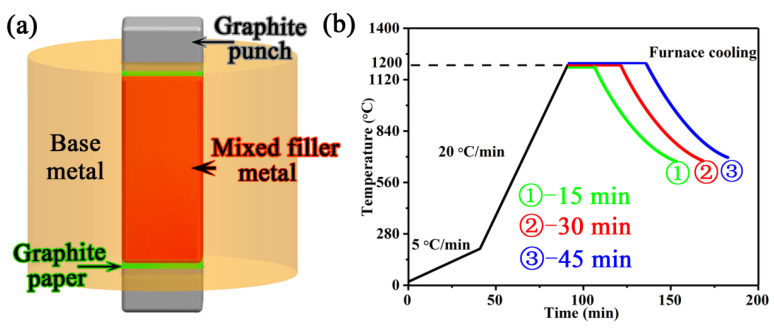
Schematic of the brazing assembly (**a**), time–temperature profile of the wide-gap brazing process (**b**).

Prior to brazing, the brazing surface of the BM was polished, and then degreased ultrasonically for 1200 s in a bath of acetone. The mixed filler powder was assembled into the wide gap in the BM and secured by upper and lower graphite punches, as shown in Figure 2a. The joint configuration was heated in an HP-12 × 12 × 12 furnace with a vacuum of ~5 × 10^−4^ Pa (Centorr Vacuum Industries, Nashua, NH, USA). The time–temperature profile of the brazing cycle is shown in Figure 2b. The specimens were brazed by heating to 1200 °C for three different time periods, namely 15, 30 and 45 min, followed by cooling in a vacuum furnace. During the wide-gap brazing process, a densifying pressure of around 20 MPa was maintained in the mixed powder-filled area. The complete brazing process is described in detail in previous reports [26].

### 2.3. Microstructure and Properties Characterization

In this study, the physical properties, phase composition, and microstructure of the mixed filler powder and as-brazed alloy were characterized by optical microscopy (OM) using a VHX-600 digital microscope (Keyence, Osaka, Japan), X-ray diffraction (XRD) analysis using a Rigaku D/MAX-2500/PC X-ray diffractometer (Rigaku Corp., Tokyo, Japan), scanning electron microscopy (SEM)/energy dispersive X-ray spectroscopy (EDS) using an FEI NOVA NANOSEM 430 scanning electron microscope (FEI Company, Hillsboro, OR, USA) equipped with an energy dispersive X-ray spectrometer, and transmission electron microscopy (TEM) using a JEM-2100F transmission electron microscope (JEOL Ltd., Tokyo, Japan).

In addition, the tensile mechanical properties of the as-brazed alloy at room temperature were characterized at a strain rate of 0.1 mm/min on a SANS CMT5105 microcomputer-controlled electronic universal testing machine (Sans Testing Machine Co. Ltd., Shenzhen, China), with tensile specimen dimensions of L60 mm × W12 mm × H3 mm. The settings of the electron microscope and positions of the tensile samples are described in detail in previous reports [26]. To ensure the accuracy and repeatability of the experiments, all tests in this study were performed in triplicate.

## 3. Results

### 3.1. Microstructure of Wide-Gap Brazed Region

**Figure 3:**
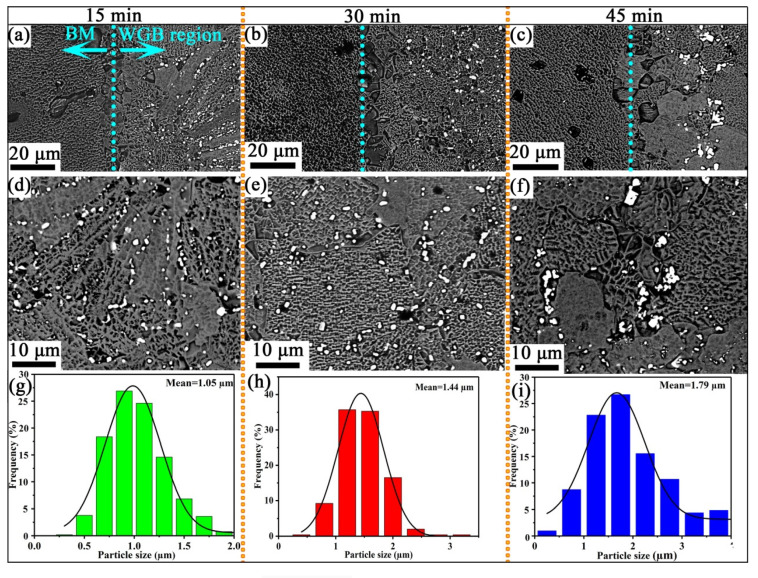
Backscattered electron micrographs of brazed joint (**a**–**c**) and wide-gap brazed (WGB) region (**d**–**f**) bonded at different brazing times. The size distribution of the M_3_B_2_ phase (**g**–**i**) in the WGB region brazed at different brazing times.

**Figure 4:**
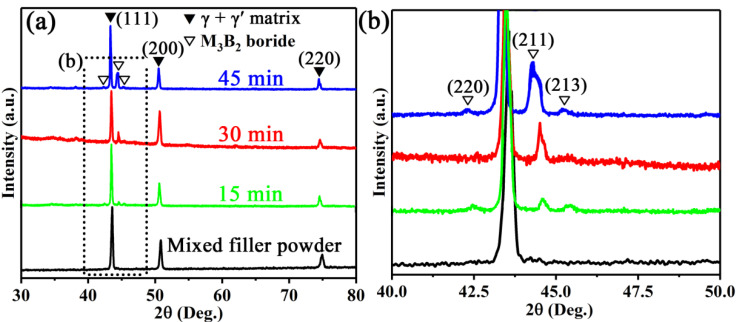
(**a**) XRD patterns of the mixed filler powder and WGB region bonded at different brazing times, (**b**) high magnification XRD patterns of 40–50 degreee in (**a**).

Similar to the results of our previous study [26], the microstructures of the brazed joints differ in the WGB region and BM, as can be seen in the micrographs shown in Figure 3a–c. The effect of the brazing time on the morphology of the WGB region was determined by performing phase measurements and backscatter electron microscopy analysis on the samples. The XRD patterns (Figure 4) of the WGB region brazed at different brazing times reveal that the phases include γ + γ′ matrix and M_3_B_2_ precipitates [27,28,29]. Moreover, the peaks of the γ + γ′ matrix and the M_3_B_2_ phase tend to be moved to the lower angles as the brazing time increases. This is due to the growth characteristics exhibited by the grains of the alloy induced by the heat accumulation with the extension of the holding time. Additionally, it was also found that the intensity of the diffraction peak of crystal plane (211) of M_3_B_2_ boride gradually increased with the extension of the brazing time, which indicates that the content of M_3_B_2_ boride gradually increased. This is mainly due to the extension of time, which provides enough time for the precipitation of M_3_B_2_ boride.

The backscattered electron micrographs of the WGB region bonded for different brazing times (Figure 3d–f) reveal significantly changed distribution, morphology, and area fraction of M_3_B_2_ particles in the corresponding WGB region with increasing brazing time (Table 2).

When the brazing time is 15 min, the M_3_B_2_ phase mainly distributed along the boundary of dendrites with chain-type shape; this may be due to the presence of higher energy in the dendrite boundary, which provides a good dynamic foundation for the precipitation of M_3_B_2_ boride [30,31].

It is worth noting that the trend towards sphericalization and equiaxing of the M_3_B_2_ boride particles in the WGB region was most obvious in the sample brazed for 30 min. When the brazing time is increased to 45 min, the M_3_B_2_ borides of the WGB region are “strip-type”, larger and distributed along the grain boundaries (Figure 3c,f), which demonstrates coarsening of M_3_B_2_ borides after an excessively long time [32].

Meanwhile, for the joint sample prepared by brazing for 30 min (Figure 3h), the M_3_B_2_ boride particles in the WGB region exhibit a relatively narrow size distribution, with most dispersed in the γ + γ′ matrix. This is mainly a result of the state of the mixed filler powder, which is more suitable for the nucleation of spherical M_3_B_2_ boride precipitates in crystals after 30 min.

This finding showed that at 30 min, the braze powder was completely melted, the mixed filler powder in the WGB region was in a homogeneous compositional state, and there was better nucleation of the M_3_B_2_ precipitates in the crystal. Overall, this further demonstrated that this brazing condition (30 min) is better for sufficient diffusion of boron into the matrix and the solid solution effect of γ + γ′ phase. [33].

### 3.2. Mechanical Properties

**Figure 5:**
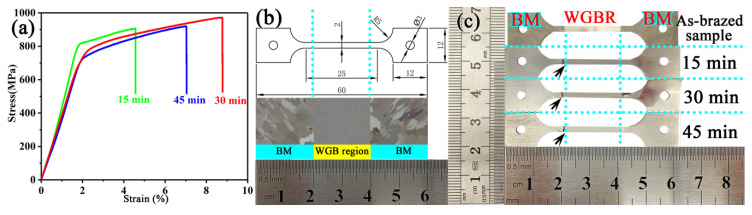
Tensile stress–stain curves of the as-brazed specimens bonded for different times (**a**), the illustration of the brazing process of the K417G alloy tensile test specimen (**b**), and morphology of failed specimens (**c**) (BM—base metal, WGBR—wide-gap brazed region).

In order to further characterize the mechanical properties of the as-brazed samples, the ultimate tensile strength (UTS) of the joints brazed for different brazing times were tested at room temperature, as shown in Figure 5. The results of the room temperature tests shown in Figure 5a reveal that the tensile strength and ductility of the repaired joint initially increased and then decreased, with the increase of the brazing time. Three tensile mechanical properties have the same trend, which indicates the result is repeatable. When the brazing time was increased to 30 min, the UTS was noticeably improved to 971 MPa, exceeding the UTS (935 MPa) of the BM. Fracture failure occurred at one side of the BM for the samples prepared at 30 min, but failure for other samples occurred at the bonding layer (Figure 5c, indicated by a black arrow). This was attributed to the reduction in the porosity and morphology, as well as the M_3_B_2_ precipitated phase in the WGB region. However, there was a reduction in the UTS of the joint after brazing for 45 min. The decrease of the bonding strength with increasing brazing time could be attributed to the larger and elongated M_3_B_2_ boride particles in the WGB region. The improvement of the strength of the joint was attributed to the shape, size, and distribution of the M_3_B_2_ boride particles in the WGB region. Fortunately, for the samples brazed at 1200 °C for 30 min, the size of the globular M_3_B_2_ boride particles is relatively concentrated, with most distributed in the γ + γ′ matrix phases [34,35]. Therefore, it is reasonable to infer that the M_3_B_2_ borides play an important role in the tensile properties of the joints during deformation.

## 4. Discussion

According to the above experimental results, for the brazed alloy with brazing time of 15 and 45 min, the fracture position is near the joint of the WGB region and BM, while only for the alloy with a brazing time of 30 min the BM breaks. These results are consistent with the mechanical properties trend observed for bonded alloys and show that the tensile strength of the brazed joint is related to the position, shape, and size of the M_3_B_2_ phase in the WGB region. Overall, these findings further demonstrated that this brazing time (30 min) is the most appropriate for the diffusion of boron into the matrix [36,37,38]. The samples brazed for 30 min have a moderate amount of spherical M_3_B_2_ boride in the WGB region, and these particles are homogeneous in size and evenly distributed in the WGB region, allowing for reduced stress concentrations during the deformation process, thereby facilitating an efficient repair [39].

**Figure 6:**
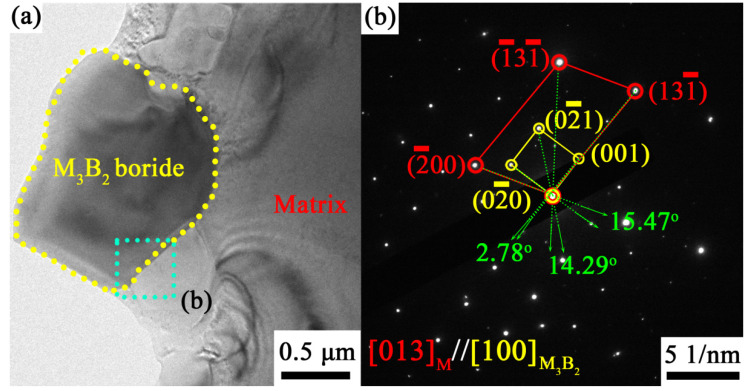
TEM micrographs (**a**) and corresponding selected area electron diffraction (SAED) patterns of phase interface (**b**) of the matrix (M-γ + γ′) and M_3_B_2_ borides in the specimen brazed for 30 min.

Analysis of the interface of the precipitated phase M_3_B_2_ and the matrix using selected area electron diffraction (SAED) (Figure 6) reveals that the crystal plane of the M_3_B_2_ precipitate [100] is parallel to that of the matrix  [013]. The orientation relationship of the M_3_B_2_ borides and the matrix can be summarized as: ${\left(13\bar{1}\right)}_{M}$//${\left(001\right)}_{M_{3}B_{2}}$, ${\left(\bar{2}00\right)}_{M}$//${\left(0\bar{2}0\right)}_{M_{3}B_{2}}$, and $\;{\left(\bar{1}3\bar{1}\right)}_{M}$//${\left(0\bar{2}1\right)}_{M_{3}B_{2}}$, respectively. For the as-brazed specimens with a brazing time of 30 min (Figure 6b), the angles of the crystal planes corresponding to the phase relationship of the M_3_B_2_ borides and the matrix are 2.78°, 15.47° and 14.29°. According to the Bramfitt lattice matching theory [40], the mismatch degree factor $\;\bar{\delta_{{\left(hkl\right)}_{M_{3}B_{2}}}^{{\left(hkl\right)}_{M}}}$ was calculated as 7.79%, and the specific parameters are listed in Table 3.

For the sample prepared at 1200 °C for 30 min, the M_3_B_2_ boride precipitates, and the matrix maintain semi-coherency, with a mismatch degree of only 7.79%. Surprisingly, after 3% pre-deformation, the mismatch degree of the M_3_B_2_ boride precipitates and the matrix in the WGB region change to 4.70% with a better coherence for the sample brazed at 1200 °C for 30 min [26]. This finding indicates that the exerted strain induces a self-accommodation effect of phase interfaces, thereby transforming the semi-coherent interface in the sample into a coherent interface in the as-brazed sample during 3% pre-deformation. This suggests a transformation into a more stable state, consequently resulting in higher strength and larger plasticity for the metallic materials [41].

According to the coherent strain strength theory model of the precipitated phase established by Mott and Nabarro [42], the fine M_3_B_2_ precipitates in the γ + γ′ matrix cause lattice misfit when deformation occurs, especially for the precipitated phase of M_3_B_2_ boride (a = b = 0.578 nm, c = 0.313 nm) and its matrix (a = b = c = 0.356 nm). As mentioned above, due to the self-accommodation effect of phase interfaces, the elastic stress field generated by the lattice mismatch will cause the lattice of the γ + γ′ matrix, whose cell volume is relatively small, to expand. As a result, it will induce the formation of numerous dislocations in the γ + γ′ matrix during the deformation process, especially near the M_3_B_2_ precipitated particles. At this point, the dislocation movement will not directly pass through these precipitated particles but will result in multiple coherent strains that hinder their motion by dislocation tangles to achieve the reinforcement effect [43].

## 5. Conclusions

In this study, a wide gap in the K417G alloy was repaired at 1200 °C by vacuum hot-pressing for different brazing times. The microstructure evolution and mechanical properties of the WGB region were thoroughly studied. The following findings were made:

(1) With the increase of the brazing time from 15 to 45 min, the tensile strength of the repaired joint increased initially and then decreased. The maximum UTS value of the joint was obtained by brazing for 30 min and was 971 MPa at room temperature, which was higher than that of the cast BM.
(2) At the brazing times of 15 min, the M_3_B_2_ boride particles were mainly distributed at the dendrite boundary, and their size was fine. The M_3_B_2_ boride particles in the joint brazed for 30 min exhibited the most uniform size and semi-coherently precipitated in the matrix, with an approximate spherical size of about ~1.44 μm. When the brazing time was further increased to 45 min, the grains and particles coarsened. Therefore, 30 min was determined to be the optimal brazing time to obtain high performance.
(3) The improvement of the strength of the K417G alloy brazed for 30 min is mainly due to the coherent strain strengthening between the dispersed M_3_B_2_ particles and its γ + γ′ matrix, as well as the mirror-deformation resistance caused by the inconsistency of the deformation between the hard particle and the soft matrix.

## Figures and Tables

**Table 1:** Chemical compositions (in wt.%) of the base metal, additive powder, braze powder, and mixed filler powder.

Materials	Co	Cr	Ti	Al	Mo	W	Nb	Zr	V	B	Ni
Base metal	10.20	8.80	4.70	5.30	3.40	/	/	/	0.80	/	Bal.
Additive powder	7.50	14.50	4.40	3.80	4.30	2.90	1.50	/	/	/	Bal.
braze powder	18.80	12.60	/	3.50	/	/	/	8.80	/	2.80	Bal.
Mixed filler powder	8.10	14.40	4.18	3.79	4.09	2.76	1.43	0.44	/	0.14	Bal.

**Table 2:** Summary of location, shape, and area fraction of M_3_B_2_ borides in the WGB region.

Brazing Time	Location	Shape	Area Fraction
15 min	Dendrite boundary	Chain-type	1.78%
30 min	Grain/Grain boundary	Globular/Blocky	2.44%
45 min	Grain boundary	Strip-type	2.82%

(Note, M_3_B_2_ particles in the electron micrographs were counted and measured by Photoshop 7.0 and Image Pro 4.5.).

**Table 3:** Matching degree factor of the phase interface between matrix and M_3_B_2_ borides.

${\left[\mathrm{uvw}\right]}_{M\;}$	${\left[\mathrm{uvw}\right]}_{M_{3}B_{2}}$	$d_{{\left[uvw\right]}_{M}}(\mathrm{nm})$	$d_{{\left[uvw\right]}_{M_{3}B_{2}}}(\mathrm{nm})$	θ (°)	$\delta_{{\left(hkl\right)}_{M_{3}B_{2}}}^{{\left(hkl\right)}_{M}}(\%)$	$\bar{\delta_{{\left(hkl\right)}_{M_{3}B_{2}}}^{{\left(hkl\right)}_{M}}}(\%)$
[$\bar{2}00$]	[$0\bar{2}0$]	0.1782	0.2924	15.47	17.47	7.79
[$\bar{1}3\bar{1}$]	[$0\bar{2}1$]	0.1103	0.2175	14.29	1.71	
[$13\bar{1}$]	[001]	0.1086	0.3123	2.78	4.20	
